# Kinetic characterisation of Erv1, a key component for protein import and folding in yeast mitochondria

**DOI:** 10.1111/febs.15077

**Published:** 2019-10-16

**Authors:** Xiaofan Tang, Swee Kim Ang, Efrain Ceh‐Pavia, Derren J. Heyes, Hui Lu

**Affiliations:** ^1^ School of Biological Sciences Faculty of Biology, Medicine and Health University of Manchester UK; ^2^ School of Materials University of Manchester UK; ^3^ Manchester Institute of Biotechnology University of Manchester UK

**Keywords:** enzyme kinetics, MIA pathway, sulfhydryl oxidase

## Abstract

Yeast (*Saccharomyces cerevisiae*) essential for respiration and viability 1 (Erv1; EC number http://www.chem.qmul.ac.uk/iubmb/enzyme/1/8/3/2.html), a member of the flavin adenine dinucleotide‐dependent Erv1/ALR disulphide bond generating enzyme family, works together with Mia40 to catalyse protein import and oxidative folding in the mitochondrial intermembrane space. Erv1/ALR functions either as an oxidase or cytochrome *c* reductase by passing electrons from a thiol substrate to molecular oxygen (O_2_) or cytochrome *c*, respectively. However, the substrate specificity for oxygen and cytochrome *c* is not fully understood. In this study, the oxidase and cytochrome *c* reductase kinetics of yeast Erv1 were investigated in detail, under aerobic and anaerobic conditions, using stopped‐flow absorption spectroscopy and oxygen consumption analysis. Using DTT as an electron donor, our results show that cytochrome *c* is ~ 7‐ to 15‐fold more efficient than O_2_ as electron acceptors for yeast Erv1, and that O_2_ is a competitive inhibitor of Erv1 cytochrome *c* reductase activity. In addition, Mia40, the physiological thiol substrate of Erv1, was used as an electron donor for Erv1 in a detailed enzyme kinetic study. Different enzyme kinetic *k*_cat_ and *K*_m_ values were obtained with Mia40 compared to DTT, suggesting that Mia40 modulates Erv1 enzyme kinetics. Taken together, this study shows that Erv1 is a moderately active enzyme with the ability to use both O_2_ and cytochrome *c* as the electron acceptors, indicating that Erv1 contributes to mitochondrial hydrogen peroxide production. Our results also suggest that Mia40‐Erv1 system may involve in regulation of the redox state of glutathione in the mitochondrial intermembrane space.

**Erv1:**

EC number http://www.chem.qmul.ac.uk/iubmb/enzyme/1/8/3/2.html.

## Graphical abstract

The *Saccharomyces cerevisiae* enzyme essential for respiration and viability 1 (Erv1) functions as an oxidase or cytochrome *c* reductase and works with Mia40 to catalyse protein import and oxidative folding in the mitochondrial intermembrane space. Here, we find that Erv1 can function both as an oxidase (using O_2_ as an electron donor) and as a cytochrome *c* reductase, depending on environmental conditions. Our findings also indicate that Mia40 modulates Erv1 enzyme kinetics and that Mia40‐Erv1 regulate glutathione’s redox state in the mitochondrial intermembrane space.

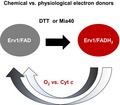

**Abbreviations**
ALR: augmenter of liver regeneration
ER: endoplasmic reticulum
Erv: essential for respiration and viability
ETC: electron transport chain
FAD: flavin adenine dinucleotide
IMS: intermembrane space
MIA: mitochondrial import and assembly

## Introduction

Disulphide bonds are required for the correct folding and function of many proteins. The process is catalysed by dedicated cellular sulfhydryl oxidoreductase systems including those of the periplasmic space of prokaryotes, the endoplasmic reticulum (ER) and mitochondrial intermembrane space (IMS) of eukaryotes 1, 2. Essential for respiration and viability 1 (Erv1), called augmenter of liver regeneration (ALR) in mammals, is an essential component of the mitochondrial IMS Mia40‐Erv1 sulfhydryl oxidoreductase system in the mitochondrial import and assembly (MIA) pathway 3, 4, 5.

Mitochondria are double‐membraned organelles, often described as the powerhouse of eukaryotic cells, playing essential roles in all important biological processes. Mitochondrial dysfunction is associated with ageing and implicated in many diseases, including cancer, diabetes and various neurodegenerative diseases. Mitochondrial biogenesis is strictly dependent on the organelle import of proteins, as the majority of mitochondrial proteins are nuclear‐encoded and synthesised in the cytosol 6, 7. All the mitochondrial IMS proteins are synthesised in the cytosol and most of them are imported into the mitochondria via thiol‐disulphide redox regulated MIA pathway 8, 9, 10. The mitochondrial MIA pathway is well‐studied using yeast *Saccharomyces cerevisiae* as a model. The pathway consists of two essential components: a disulphide bond carrier (oxidoreductase) Mia40 and a flavin adenine dinucleotide (FAD)‐dependent disulphide bond generator Erv1/ALR. Most substrates of the MIA pathway contain conserved cysteine residues, which are maintained in a reduced form in the cytosol and subsequently oxidised to form disulphide bonds in the mitochondrial IMS 11, 12, 13. Upon mitochondrial import, Mia40 interacts with its substrate proteins directly and transfers a disulphide bond to the substrates via formation of intermolecular disulphide linked complexes 14, 15. The reduced Mia40 is then reoxidised by Erv1/ALR for regeneration 15, 16.

**Figure 1:**
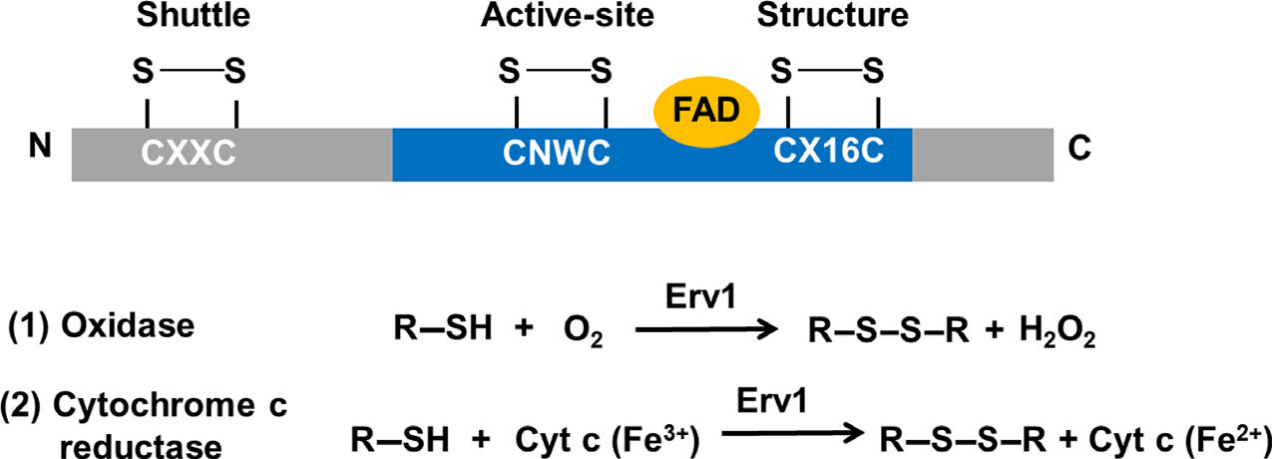
Structure and reactions catalysed by Erv1/ALR enzymes. (Top) A schematic structure and the Cys motifs of Erv1: The highly conserved FAD‐binding (catalytic) domain of Erv1 is shown in black, containing the redox‐active CXXC disulphide and structural CX16C disulphide, while the unfolded regions are in grey. Both yeast Erv1 and human ALR enzymes have a shuttle CXXC motif at the nonconserved N‐terminal domain. (Below) The reactions catalysed by Erv1/ALR as oxidase (1) and cytochrome *c* reductase (2), respectively.

Essential for respiration and viability 1/ALR homologs have been identified in all mitochondria containing eukaryotes, playing an essential role in cell viability 17, 18. They all possess a highly conserved FAD‐binding domain catalytic core with an active‐site CXXC disulphide bond located proximal to the isoalloxazine ring of the FAD cofactor and a CX_16_C structural disulphide (Fig. 1). Erv1 of *S. cerevisiae* (yeast Erv1) has the FAD‐binding domain at the C‐terminus together with the active‐site (Cys130–Cys133) and structural (Cys159–Cys176) disulphide bonds 16. Both yeast and human enzymes also have a shuttle disulphide bond at the nonconserved N‐terminal domain (Cys30–Cys33 in Erv1).We showed previously that Erv1 can be reduced by reduced Mia40 or disulphide reducing agents, for example, DTT and tris(2‐carboxyethyl)phosphine, via reduction of the shuttle disulphide 16, 19. The electrons are transferred to the active‐site disulphide, the cofactor FAD, and lastly the reduced Erv1 can be reoxidised by transferring electrons to molecular oxygen (O_2_) directly (acting as a sulfhydryl oxidase) or to cytochrome *c* (acting as a cytochrome *c* reductase) for regeneration 20, 21. However, the relative substrate specificity of Erv1 for oxygen and cytochrome *c* is unknown.

Cytochrome *c* is a highly conserved haemoprotein located in the mitochondrial IMS and a key component of the mitochondrial electron transport chain (ETC). Cytochrome *c* transfers electrons from ubiquinone–cytochrome *c* oxidoreductase (COX, complex III) to cytochrome *c* oxidase (complex IV) during respiration. Using DTT as an electron donor, Farrell and Thorpe 22 showed that horse heart cytochrome *c* is ~ 100‐fold better than O_2_ as an electron acceptor for the short form of human ALR (sfALR), and suggested that cytochrome *c* was a biologically preferred substrate of ALR. On the other hand, *Trypanosoma brucei* Erv1 (TbErv1) was shown to reduce O_2_ and cytochrome *c* at a similar activity, with no obvious preference for cytochrome *c* 23. Moreover, there was about 40% decrease in the rate of cytochrome *c* reduction under aerobic compared with anaerobic conditions, suggesting that O_2_ may even be the preferred electron acceptor for TbErv1 23. As for yeast Erv1, it was suggested that cytochrome *c* was especially important under oxygen‐limiting conditions 24, a preferred substrate *in vivo* for yeast Erv1 20. However, the relative substrate specificity was not compared directly through detailed enzyme kinetic study.

In this study, we performed a detailed enzyme kinetic study to understand the catalytic efficiency of yeast Erv1 for O_2_ and cytochrome *c*. Oxygen consumption analysis and stopped‐flow measurements to follow cytochrome *c* reduction under both aerobic and anaerobic conditions were also performed. This study can be divided into two main parts. Firstly, DTT, a commonly used chemical electron donor of sulfhydryl oxidases, was used for Erv1 enzyme kinetic studies, and the results were compared with those of human sfALR 22 and TbErv1 23. Secondly, although Mia40 was used previously in various reconstituted activity assays for Erv1 and indicated that Mia40 may be more efficient than DTT 17, 25, 26, 27, 28, in this paper, Mia40 was used for the first time for a detailed enzyme kinetic study to understand and verify the difference in relative oxidase and cytochrome *c* reductase activity of Erv1/ALR enzymes. Taken together, this study showed that Erv1 is a moderately active enzyme with the ability to use both O_2_ and cytochrome *c* as the electron acceptors. Furthermore, our data also suggest that Mia40 participates in mediating Erv1 enzyme kinetics.

## Results

### Cytochrome *c* reductase activity of Erv1 using DTT as an electron donor

**Figure 2:**
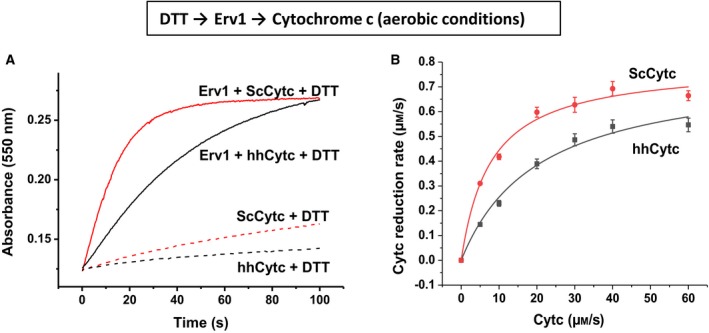
Cytochrome *c* reductase activity of yeast Erv1. (A) Time courses of cytochrome *c* (10 μm ScCytc or hhCytc) reduction by 1 mm DTT in the presence or absence of 1 μm Erv1. The reaction was initiated using stopped‐flow fast mixing technique and followed by measuring the absorbance change at 550 nm. (B) Cytochrome *c* (hhCytc in grey, ScCytc in black) concentration dependence of the Erv1 catalysed reaction. The initial rates (after subtraction of nonenzymatic background rate) were fitted with the Michaelis–Menten equation (solid lines). The kinetic parameters were summarised in Table 1. The error bars represent standard error of mean (SEM), *n* = 3.

The horse heart cytochrome *c* (hhCytc) is the most commonly used model cytochrome *c* because it is commercially available, cheap and stable in its oxidised form. We first assessed if there is a major difference between hhCytc and yeast cytochrome *c* (ScCytc), which share about only ~ 63% sequence identity, as a substrate of Erv1. As shown in Fig. 2A, whilst hhCytc is more stable against reduction by DTT, reduction of both forms of cytochrome *c* was catalysed by Erv1 based on the increase of absorbance at 550 nm. Detailed enzyme kinetic analyses, at various concentrations of hhCytc or ScCytc, were performed and Michaelis–Menten analyses were performed (Fig. 2B). The results showed that the two forms of cytochrome *c* have the same *k*_cat_ values of 0.82 ± 0.07 s^−1^, but different *K*_m_ values of 24 ± 4 μm for hhCytc and 12 ± 3 μm for ScCytc (Fig. 2; Table 1). The substrate specificity or catalytic efficiency (*k*_cat_/*K*_m_) was 3.6 × 10^4^ m ^−1^·s^−1^ and 6.7 × 10^4^ m ^−1^·s^−1^, respectively. Thus, although ScCytc is intrinsically less stable than hhCytc against DTT reduction and has only 63% primary sequence identity, reduction of both proteins showed the same turnover number (*k*_cat_), but ScCytc had slightly lower *K*_m _and thus slight higher substrate specificity or catalytic efficiency (*k*_cat_/*K*_m_) for yeast Erv1 (Table 1).

**Table 1:** Cytochrome *c* reductase kinetics of yeast Erv1 determined under aerobic or anaerobic conditions, at 25 °C, pH 7.4.

	Electron donor	Electron acceptor	*k*_cat_ (s^−1^)	*K*_m_ (μm)	*k*_cat_/*K*_m_ (m ^−1^·s^−1^)
Aerobic	DTT	hhCytc	0.82 ± 0.07	23 ± 4	3.6 × 10^4^
	DTT	ScCytc	0.82 ± 0.05	12 ± 3	6.7 × 10^4^
Anaerobic	DTT	hhCytc	0.75 ± 0.04	11 ± 2	6.8 × 10^4^
	DTT	ScCytc	0.75 ± 0.02	5 ± 2	1.5 × 10^5^
	Mia40	ScCytc	8.5 ± 0.2	25 ± 2	3.3 × 10^5^

### Effect of oxygen on the cytochrome *c* reductase activity of Erv1

**Figure 3:**
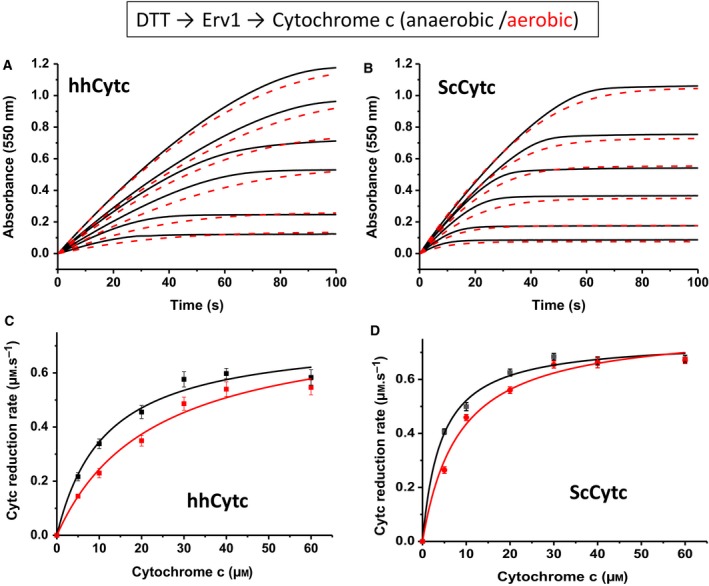
Cytochrome *c* reductase activity of yeast Erv1 under anaerobic conditions. (A) Reduction at various concentrations (5–60 μm) of hhCytc by 1 mm DTT as measured by following the absorbance change at 550 nm, in the presence of 1 μm Erv1 and 10 units·mL^−1^ of SOD. Erv1 was mixed with hhCytc in one syringe before mixing with DTT and SOD in another syringe using stopped‐flow equipment located inside an anaerobic chamber (O_2_ < 2 p.p.m.; black lines). For comparison, the time courses of the reactions measured under normal aerobic conditions were shown (red lines). (B) As in A, but using ScCytc. (C, D) Michaelis–Menten plots of the results shown in A and B, respectively. The lines represent analysis using Michaelis–Menten equation. Error bars represent SEM, *n* = 3. All the enzyme kinetic parameters are summarised in Table 1.

To understand how oxygen affects the cytochrome *c* reductase activity of Erv1, we performed the same experiment for Erv1, but under anaerobic conditions using a stopped‐flow instrument inside an anaerobic chamber. The results were analysed using Michaelis–Menten equation and compared with those from aerobic conditions. As shown in Fig. 3 and Table 1, under anaerobic conditions both hhCytc and ScCytc have the same *k*_cat_ of about 0.75 ± 0.04 s^−1^ and again an approximately twofold difference in *K*_m _values. The *K*_m_ values are about 11 μm for hhCytc and 5 μm for ScCytc, supporting the suggestion that ScCytc is a slightly better substrate than hhCytc due to its comparatively lower *K*_m_. Comparing these results with those obtained under aerobic conditions, it is clear that the *k*_cat _values were not affected by the presence of oxygen (at about 250 μm) nor the different origins of cytochrome *c*. On the other hand, both *K*_m _values decreased by ~ 50% under the aerobic conditions. Thus, we concluded that oxygen acts as a competitive inhibitor of cytochrome *c* reductase activity of Erv1.

### Oxidase activity of Erv1 using DTT as an electron donor

**Figure 4:**
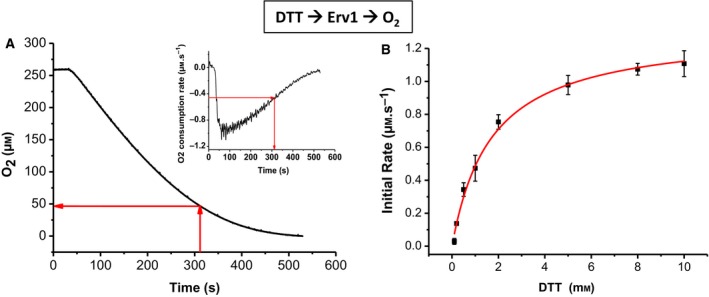
Oxidase activity of yeast Erv1. (A) Oxygen consumption curve of 5 mm DTT in the presence of 1 μm Erv1 and 10 units·mL^−1^ of SOD. Inset: the first derivative of the data, which was used to identify the point at which the rate was half maximal. *K*_m_ for oxygen was determined to be about 50 ± 10 μm, and *k*_cat_ was 1.0 ± 0.1 s^−1^. (B) Michaelis–Menten plot of Erv1 at various concentrations of DTT. *k*_cat_ and *K*_m _values of 1.28 ± 0.03 s^−1 ^and 1.5 ± 0.1 mm, respectively, were obtained. Error bars represent SEM, *n* = 3.

In this study, the oxygen consumption of DTT (5 mm) in the presence of 1 μm Erv1 and superoxide dismutase 1 (SOD1, to remove potential superoxide formed) was measured (Fig. 4A). Differentiation of the data was performed, and the Michaelis–Menten kinetic parameters *k*_cat_ and *K*_m_ were calculated to be 1.0 ± 0.1 s^−1^ and 50 ± 10 μm, respectively (Table 2). The results are similar to that we determined previously using 10 mm DTT but in the absence of SOD1 16. To estimate the catalytic efficiency or substrate specificity (*k*_cat_/*K*_m_) of Erv1 at 1 mm DTT, the DTT concentration dependence of the rate of oxygen consumption was measured (Fig. 4B). Michaelis–Menten analysis showed that *k*_cat_ and *K*_m _values for DTT were 1.28 ± 0.03 s^−1^and 1.5 ± 0.1 mm, respectively. Thus, the apparent *k*_cat_ of Erv1 for oxygen at 1 mm DTT (as for cytochrome *c* reduction assay) was calculated to be about 0.5 s^−1^ and thus substrate specificity is 1.0 × 10^4^ m ^−1^·s^−1^. A similar result was estimated based on using 1 mm DTT as the electron donor (not shown).

**Table 2:** Oxidase kinetics of yeast Erv1 determined at 25 °C, pH 7.4.

Electron acceptor	*k*_cat_ (s^−1^)	*K*_m_ (μm)	*k*_cat_/*K*_m_ (m ^−1^·s^−1^)
5 mm DTT	1.0 ± 0.1	50 ± 10	2 × 10^4^
1 mm DTT	0.5 ± 0.1	50 ± 10	1 × 10^4^
10 mm GSH + 50 μm Mia40	0.05 ± 0.01	3 ± 1	1.7 × 10^4^

To compare the relative substrate specificity (RSS) of Erv1 for cytochrome *c* and oxygen, we defined RSSc/o = [*k*_cat_/*K*_m_]^Cytc^/[*k*_cat_/*K*_m_]^oxygen^. As shown in Table 3, with 1 mm DTT as the electron donor, ScCytc is about 15‐fold more efficient than oxygen as a substrate for Erv1 under anaerobic conditions (about sevenfold better under aerobic conditions). Similar to human sfALR, Erv1 is a better cytochrome *c* reductase than oxidase. On the other hand, the RSSc/o of yeast Erv1 is not as high as that of sfALR (15 vs 107) but clearly higher than that of TbErv1 (15 vs 2).

**Table 3:** RSS (*k*_cat_/*K*_m_) of known Erv1/ALR enzymes between cytochrome *c* (under anaerobic conditions) and oxygen.

	Cytochrome *c* *k*_cat_/*K*_m_ (m ^−1·^s^−1^)	Oxygen *k*_cat_/*K*_m_ (m ^−1·^s^−1^)	RSSc/o^a^	Reference
sfALR (human)	4.5 × 10^5^	4.2 × 10^3^	107	22
Erv1 (yeast)	1.5 × 10^5^	1.0 × 10^4^	15	This study
TbErv1 (*Trypanosoma brucei*)	2.1 × 10^4^	1.2 × 10^4^	2	23

a RSSc/o = [*k*_cat_/*K*_m_]^Cytc^/[*k*_cat_/*K*_m_]^oxygen^.

### Cytochrome *c* reductase activity of Erv1 using Mia40 as an electron donor

**Figure 5:**
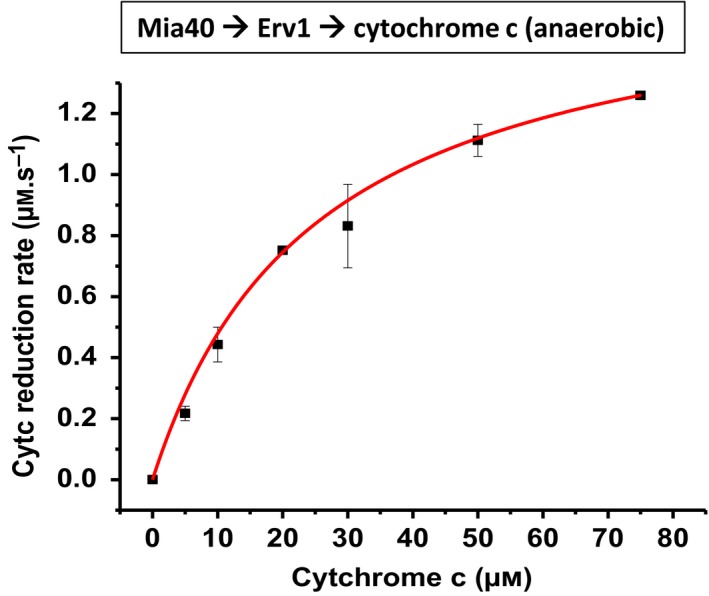
Michaelis–Menten plot of yeast Erv1 cytochrome *c* reductase activity using yeast Mia40 as the electron donor. ScCytc at various concentrations was mixed with reduced Mia40 using stopped‐flow equipment under anaerobic conditions, in the presence of 0.2 μm Erv1. The data were analysed with Michaelis–Menten equation (red line). The error bars represent SEM, *n* = 3. *K*_m_ and *k*_cat_ values were determined to be 25 μm and 8.5 s^−1^, respectively.

Though DTT is a convenient and commonly used electron donor for the activation of sulfhydryl oxidases, it is a chemical disulphide bond reducing agent that reduces disulphide bonds nonspecifically. In practice, Mia40 is the physiological electron donor or upstream substrate of Erv1/ALR enzymes in mitochondria. To study the cytochrome *c* reductase kinetics of Erv1, reduced Mia40 was prepared as described previously 29, 30. ScCytc at various concentrations was mixed with 160 μm reduced Mia40 at 1 : 1 (v/v) ratio using stopped‐flow equipment under anaerobic conditions. The absorption change at 550 nm was measured, and the data were analysed using Michaelis–Menten kinetics (Fig. 5). The *K*_m_ of cytochrome *c* was determined to be 25 μm, approximately fivefold higher than that determined with DTT. The *k*_cat_ was 8.5 s^−1^, ~ 11‐fold higher than that determined with DTT (Table 1). These results suggested that Mia40 may participate in modulating Erv1 cytochrome *c* reductase kinetics directly, as both *K*_m_ and *k*_cat_ parameters were changed. The large increase in the turnover number (*k*_cat_) indicates that Mia40 may facilitate the dissociation or release of reduced cytochrome *c* from Erv1. However, the overall catalytic efficiency (*k*_cat_/*K*_m_) of 3.3 × 10^5^ m ^−1^·s^−1^ was only about twofold higher than that determined with DTT as the electron donor, showing that there is compensation between *k*_cat_ and *K*_m_.

### Oxidase activity of Erv1 using Mia40 as an electron donor

**Figure 6:**
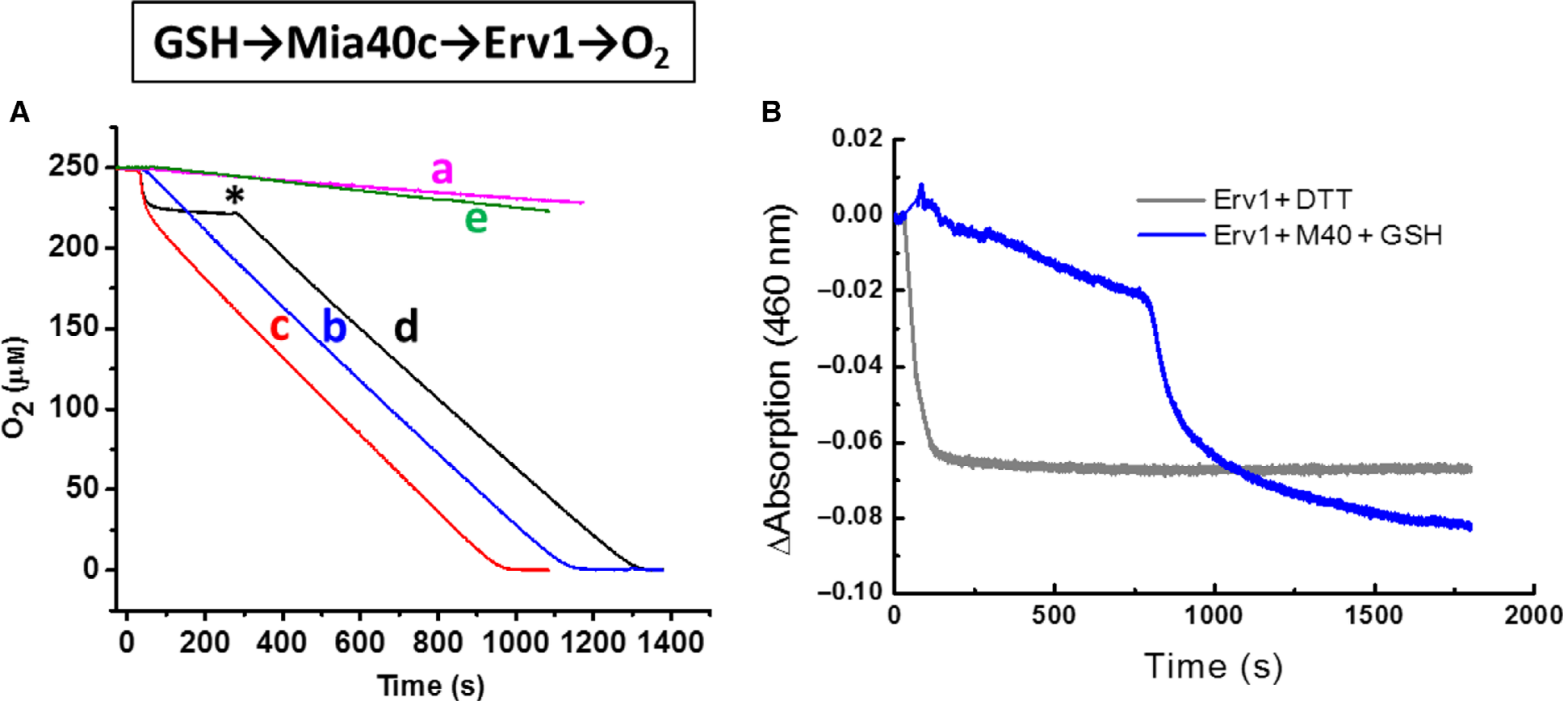
Oxidase activity of yeast Erv1 using yeast Mia40 as the electron donor. (A) Oxygen consumption in the presence of 5 μm Erv1 plus 10 mm GSH and/or 50 μm Mia40. a: Erv1 and GSH only; b: Erv1, GSH and oxidised Mia40; c: Erv1, GSH and reduced Mia40; d: Erv1, reduced Mia40 initially and followed by addition of GSH at 300 s (*); e: as b but replaced Mia40 with the Mia40SPS mutant. (B) Time‐course of absorbance change at 460 nm of Erv1 (5 μm) by 10 mm GSH and 50 μm Mia40 system (blue line) and 5 mm DTT (grey line), respectively.

Mia40 can be purified and prepared in a CPC reduced form as an electron donor for Erv1. However, it cannot be prepared at a level (mm) high enough for enzyme kinetic parameter determination, due to protein aggregation. It has been shown previously that one of the factors that affect the redox state of Mia40 is glutathione (GSH). Mia40 can be reduced by 10 mm GSH in the mitochondrial IMS 24. Hence, we performed oxygen consumption assays using Mia40 in the presence of 10 mm GSH to keep the thiol substrate concentration effectively unchanged upon oxygen depletion. As shown in Fig. 6, whilst GSH alone cannot activate Erv1 for oxygen consumption (Fig. 6A, trace a), in the presence of both GSH and oxidised Mia40 (Fig. 6A, trace b), oxygen consumption is catalysed by Erv1. Thus, Mia40 was reduced by GSH and then acted as the electron donor for Erv1. An apparent *k*_cat_ of 0.05 ± 0.01 s^−1^ and *K*_m_ value of 3 ± 1 μm for oxygen was determined (Table 2). Furthermore, in the presence of GSH and reduced Mia40 (Fig. 6A trace *c*), oxygen consumption was initially rapid and then maintained at the same rate as that of trace b (GSH plus oxidised Mia40). A similar result was obtained by sequentially adding reduced Mia40 first followed by GSH (Fig. 6A trace d), suggesting that the observed rate of the linear oxygen consumption was determined by the reduction of Mia40. It is supported by the result of the redox‐inactive Mia40SPS mutant (Fig. 6A trace e), in which no oxygen consumption was observed. The result confirmed that it was not due to the binding of Mia40 to Erv1 that allowed more efficient reduction of Erv1 by GSH directly, but that Mia40 CPC is required. Consistent with this conclusion, the time course of Erv1 absorbance change at 460 nm, at the same conditions as the oxygen consumption assay, showed that the majority of the FAD of Erv1 was in the oxidised state during oxygen consumption for about 15 min until oxygen was almost fully depleted (Fig. 6B). In contrast, 5 mm DTT efficiently reduced FAD in about 1 min. Thus, the apparent low *k*_cat_ and low *K*_m_ values for oxygen was observed for the GSH‐Mia40‐Erv1 system, and Mia40 reduction was the limiting step of the reaction. Interestingly, although the apparent *k*_cat_ and *K*_m_ values were changed about 10‐fold, an apparent oxidase catalytic efficiency (*k*_cat_/*K*_m_) of 1.7 × 10^4^ m ^−1^·s^−1^ was determined for the GSH‐Mia40‐Erv1 system, which is similar to that determined with DTT as the electron donor (Table 2).

## Discussion

**Figure 7:**
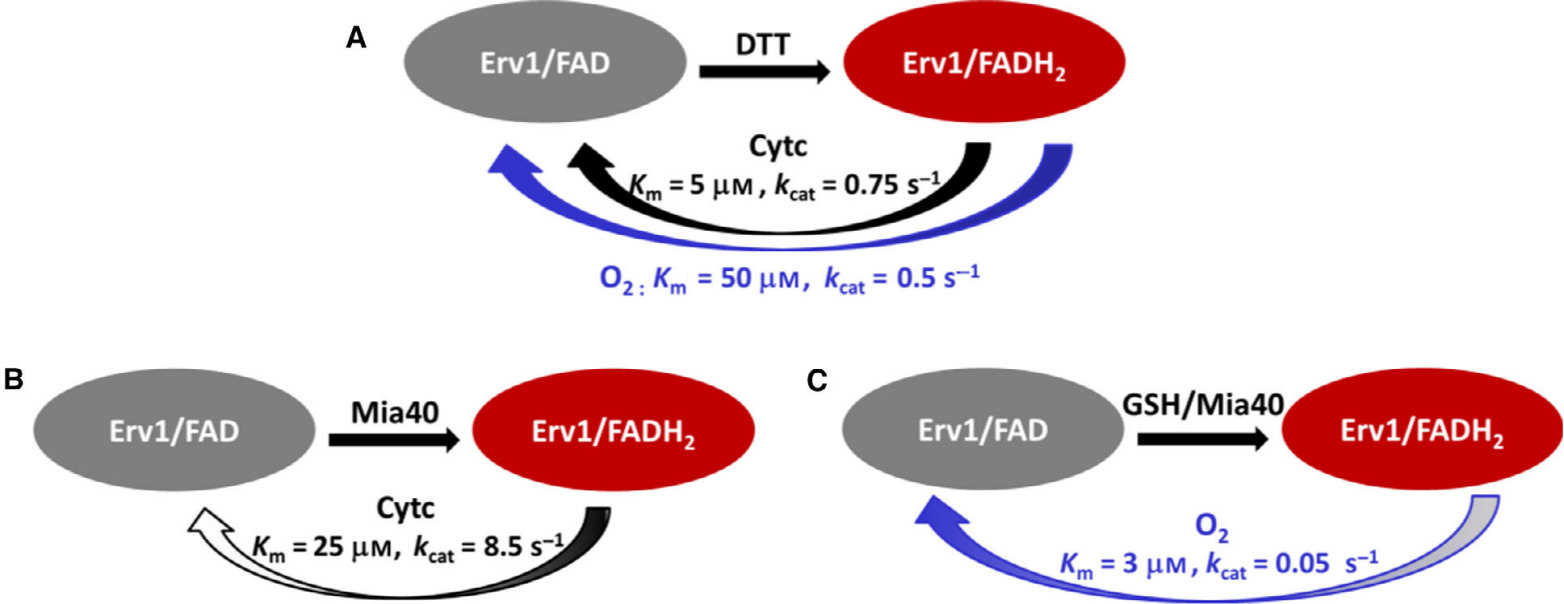
Models of yeast Erv1 oxidase and cytochrome *c* reductase activity. (A) Using DTT as a model electron donor, cytochrome *c* is more efficient than molecular oxygen as electron acceptor for Erv1 as evidenced by both low *K*_m_ and high *k*_cat_ values determined under the same conditions. (B) Using Mia40 as an electron donor for Erv1, both the *k*_cat_ and *K*_m_ values were increased for cytochrome *c* compared with those determined using DTT. (C) Using the GSH‐ Mia40 system as an electron donor for Erv1, both the *k*_cat_ and *K*_m_ values were decreased for oxygen compared with that determined with DTT. In B and C, the arrow colour changes show that *k*_cat_ in B but *K*_m_ in C contribute more positively to the catalytic efficiency compared with that in A.

In this report, we presented a detailed enzyme kinetic study to understand the oxidase and cytochrome *c* reductase activities of yeast Erv1 using both DTT and Mia40 as electron donors. The cytochrome *c* reductase kinetics was studied using stopped‐flow absorption under both aerobic and anaerobic conditions. Our results showed that Erv1, like other Erv1/ALR, is an enzyme with catalytic efficiency (*k*_cat_/*K*_m_) at a level of 10^4^–10^5^ m ^−1^·s^−1^, even with Mia40 as the thiol substrate (Tables 1, 2, 3, Fig. 7). Thus, Erv1/ALR enzymes are moderately efficient enzymes compared with those of central metabolism, such as cytochrome *c* peroxidase, fumarase and SOD, as defined by Bar‐Even et al 31. This is perhaps due to the fact that the process of protein import and/or disulphide bond formation in mitochondria is relative slow, and thus there is no requirement or evolutionary pressure to increase the Erv1/ALR catalytic efficiency. Furthermore, it may be due to the fact that Erv1 does not directly interact with newly imported substrate proteins but seems to be binding only to Mia40 as far as we know.

Our results suggest that both O_2_ and cytochrome *c* are electron acceptors of yeast Erv1. Based on the results of using DTT as an electron donor, cytochrome *c* is ~ 7–15 times more efficient than molecular oxygen depending on the oxygen tension (Tables 1 and 3, Fig. 7A). The result is consistent with human sfALR, in terms of a better cytochrome *c* reductase than oxidase 22. However, there is a clear difference in terms of the RSSc/o that is about 107 for human sfALR. This difference may be resulted by the differences in their sequences, for example, the redox‐active site CXXC motif is CEEC for ALR but CWNC for yeast Erv1. Interestingly, our results showed that the presence of Mia40 affects the *K*_m_ and *k*_cat_ values (Fig. 7). Whether Erv1 acts as an oxidase or cytochrome *c* reductase depends on the availability of oxygen and cytochrome *c* in the mitochondrial IMS. Whilst the concentration of cytochrome *c* in the mitochondria is unknown, it is believed that most cytochrome *c* molecules are bound to the mitochondrial inner membrane and associated with the phospholipid cardiolipin under normal physiological conditions. It was shown that about 85% of cytochrome *c* is located together with the ETC complexes in the cristae and about 15% of the protein is available in the IMS 32, 33. How much cytochrome *c* is located in an oxidised form in the IMS available for Erv1/ALR is unknown and it may vary widely depending on cell growth conditions. Whilst the exact concentration of O_2_ in yeast mitochondrial IMS is also unknown, it is reasonable to assume that as a single‐cell organism the concentration may be close to that of the environment. Moreover, yeast and many other cell types do not need the mitochondrial ETC for energy (ATP) production and can instead rely solely on glycolysis for energy. However, such cells still need mitochondria for other functions, such as iron‐sulfur cluster biogenesis. Thus, the oxidase function of Erv1 or GSH‐Mia40‐Erv1 system may play an important role during biogenesis of mitochondrial IMS proteins under those conditions.

Most studies on Erv1/ALR sulfhydryl oxidases have been performed using chemical reducing agents, such as DTT, as electron donors. This is the first study comparing artificial reagents with Mia40, the physiological electron donor of Erv1/ALR, in assessing the enzyme kinetics of Erv1. An interesting finding is that Mia40 seems to participate in mediating the cytochrome *c* reductase activity of Erv1, as evidenced by the obvious increase of both *K*_m,_ and *k*_cat_ values of Erv1 compared with those determined using DTT as the electron donor (Fig. 7). The result indicates that Mia40 facilitates the dissociation of reduced cytochrome *c* from Erv1, thus increasing the turnover of the enzyme for reuse. However, this is compensated by a decreased affinity for oxidised cytochrome *c*.

Another important finding is that the GSH‐Mia40‐Erv1system has a high affinity for oxygen with a *K*_m_ of 3 μm, similar to that of ER sulfhydryl oxidase Ero1 34, 35. The *K*_m_ of Ero1, the ER resident sulfhydryl oxidase enzymes, was determined to be 4 μm for yeast Ero1 and 5 μm for mammalian Ero1‐Lα 34, 35. Interestingly, a recent study showed that Ero1not only has a high affinity for molecular oxygen but also a high cooperativity of oxygen binding, whilst yeast Erv1 has no such cooperativity 36. Moreover, using GSH‐PDI (protein disulphide isomerase) as electron donor, a *K*_m_ of 89 μm and *k*_cat_ of 0.41 s^−1^ for Erv1 oxidase activity was obtained, which are both clearly larger than that we obtained with GSH‐Mia40 system (89 vs 3 μm; 0.41 vs 0.05 s^−1^). The comparison between these results provided another support for our conclusion that Mia40 plays a role in mediating the enzyme kinetics of Erv1.

The precise concentration of GSH in the mitochondrial IMS is unknown, but was suggested to be similar to that in the cytosol of about 13 mm 37. Thus, Mia40 can be reduced by GSH in the IMS at physiological relevant concentrations. Interestingly, whilst 10 mm GSH can reduce Mia40 and thus activate Erv1, a low concentration of GSH, for example, 5 mm, cannot reduce Mia40 and thus activate Erv1 (data not shown). Taken together, our results suggest that yeast Erv1 can function as an oxidase at low oxygen tension to help maintain Mia40 in an oxidised form, and may also be involved in regulation of the redox state of GSH in mitochondrial IMS.

The apparent low *K*_m_ of GSH‐Mia40‐Erv1 for oxygen suggests that the reduction of oxygen to hydrogen peroxide (Fig. 1) could be an important route for electron transfer during the import of the IMS proteins and/or redox regulation in the mitochondrial IMS. When O_2_ is used as an electron acceptor, each pair of disulphide bond formed in a substrate protein will be accompanied by the formation of hydrogen peroxide (H_2_O_2_). Hydrogen peroxide is one of reactive oxygen species (ROS) which has the potential to damage all biomolecules, including proteins, DNA and membranes. A high level of ROS can cause oxidative stress, cellular damage and has been linked to many diseases and aging 38. On the other hand, ROS are produced under normal physiological conditions and play a regulatory role in cellular metabolic processes. Under normal aerobic conditions, mitochondria are the main endogenous source of cellular ROS, as a result of incomplete electron transfer along the ETC 39. ROS can also be formed from enzyme‐catalysed reactions, such as mitochondrial protein biogenesis via the MIA pathway. Clearly, cells need to develop strategies to deal with the production of ROS by mitochondria.

Interestingly, different organisms seem to use different approaches in controlling H_2_O_2_. In yeast, H_2_O_2_ produced in the mitochondrial IMS can be subsequently reduced to water by cytochrome *c* peroxidase (Ccp1)^21^. H_2_O_2_ may also be removed by the IMS localised GSH peroxidase Gpx3 especially under oxidative stress conditions 40. Both of these peroxidase enzymes were shown to interact with components of the MIA machinery directly, thus they can scavenge hydrogen peroxide produced from the MIA pathway effectively. Moreover, a recent study showed that yeast Erv1 can also transfer electrons to the IMS localised fumarate reductase Osm1, and via Osm1 transfer electrons to fumarate under both aerobic and anaerobic conditions 41. Thus, yeast can exploit diverse electron acceptors for MIA pathway in the IMS, with at least three electron acceptors, oxygen, cytochrome *c* and Osm1/fumarate, have been identified. Interestingly, no homologues of Ccp1 and Osm1 have been found in higher eukaryotes, reflecting the fact that human sfALR prefers cytochrome *c* much more than oxygen as an electron acceptor compared with yeast Erv1 and TbErv1 (Table 3). It is also possible that higher eukaryotes have developed currently unknown alternative strategies to remove or use the H_2_O_2_ produced from the MIA pathway.

## Materials and methods

### Materials

All chemicals used in this study were of analytical grade and obtained from Sigma Aldrich Inc. (St. Louis, MO, USA) or Thermo Fisher Scientific (Waltham, MA USA) unless specified. All solutions were prepared using MilliQ water (Barnstead™ Nanopure™, Thermo Fisher Scientific). All experiments were carried out in a buffer called BAE (50 mm Tris/HCl, 150 mm NaCl, 1 mm EDTA, pH 7.4) unless specified. Bovine erythrocyte SOD1 (a Cu‐Zn‐SOD) was purchased from Sigma‐Aldrich Inc. (cat no: 9054‐89‐1).

### Protein expression and purification

The WT and mutants of Erv1 proteins were expressed and purified as previously described 29, 30. Briefly, the pET‐24a(+) plasmid containing the ERV1 gene was expressed in *Escherichia coli* Rosetta‐gami^TM ^2 cells (Sigma‐Aldrich Inc). Cells were grown in LB medium at 37 °C until OD600 of 0.3–0.4 was achieved and then proteins were induced by addition of 0.5 mm IPTG (Formedium Ltd., Swaffham, UK) at 16 °C overnight. The cell lysate was centrifuged and the proteins were purified from the supernatant using Ni‐NTA (Ni^2+^‐nitrilotriacetate) His‐Bind beads (Sigma‐Aldrich Inc). Further purification was performed using size‐exclusion chromatography using BAE and a Superdex 200 100/300 GL column (GE Healthcare Bio‐sciences, Uppsala, Sweden). An extinction coefficient of 12.3 mm ^−1^·cm^−1^ at 460 nm was used to calculate concentration of Erv1. Mia40 (the C‐terminal domain of Mia40, residues 284–403), were expressed and purified as described previously 29, 42. The hhCytc (cat. 9007‐43‐6) and ScCytc (cat. 9007‐43‐6) were purchased from Sigma‐Aldrich Inc. Their redox states were checked based on their absorption to be > 95% in the oxidised forms and were used as purchased.

### Oxygen consumption assays

Essential for respiration and viability 1 enzymatic activity towards oxygen was measured using a Clark‐type oxygen electrode (Hansatech Instruments Ltd., Norfolk, UK) in a 0.5 mL of reaction volume at 25 °C in BAE as previously described 29, 30. When DTT was used as electron donor, bovine erythrocyte SOD1 (Sigma‐Aldrich Inc., cat no: 9054‐89‐1) was added at 10 unit·mL^−1^ to exclude the potential interference of superoxide ion 43. For Erv1 oxidase kinetic perimeter determination, 5 mm DTT was used so that DTT concentration was more than 10‐fold excess than O_2_ concentration (~ 250 μm). The rate of oxygen consumption was calculated by data differentiation using originpro software (OriginLab, Northampton, MA, USA). At least three experiment repeats for each experiment were performed.

### Cytochrome *c* reduction assay

For anaerobic assays, cytochrome *c* reduction experiments were performed using an SX20 stopped‐flow instrument (Applied Photophysics, Surrey, UK) inside an anaerobic glove box with oxygen levels maintained below 2 p.p.m. BAE was made anaerobic by extensive bubbling with oxygen‐free nitrogen followed by incubation inside the anaerobic glove box over 48 h. Erv1 and chemicals were buffer‐exchanged into anaerobic BAE using PD10 column. For aerobic assays, the same experiments were repeated under aerobic condition with the same stopped‐flow instrument but with glove box opened and oxygen saturated to ambient condition (about 250 μm). In each set of DTT experiment, DTT and SOD stock solution was put in one syringe (DTT 2 mm and SOD 20 units) and Erv1 (2 μm) mixed with various concentrations of cytochrome *c* (10–120 μm) were put in the other syringe. A driving piston then pushes both syringes simultaneously into the mixer at 1 : 1 (v/v) ratio, and the absorbance change at 550 nm was measured inside a measurement cell. For experiments with Mia40, ScCytc at various concentrations and Erv1 (0.4 μm Erv1) in one syringe was mixed with 160 μm reduced Mia40 in the other syringe at 1 : 1 (v/v) ratio using stopped‐flow equipment under anaerobic conditions. The extinction coefficients ε550 of 29.5 mm ^−1·^cm^−1^ for oxidised cytochrome *c* and 8.4 mm ^−1·^cm^−1^ for reduced cytochrome *c* were used. The reduction rate of cytochrome *c* was calculated using ∆ε550 of 21.1 mm ^−1·^cm^−1^, the extinction coefficient difference between reduced and oxidised cytochrome *c*. The rate of nonenzyme catalysed cytochrome *c* reduction by DTT was measured in the absence of Erv1, which was used as background rate and subtracted from the observed rate of reduction in the presence of Erv1. The data were analysed using originpro software. At least three experimental repeats were performed for all experiments.

## Conflicts of interest

The authors declare no conflict of interest.

## Author contributions

All authors have contributed to experimental design, data analysis and manuscript writing. XT performed the experiments for Figs 2, 3, 4 and 6B; SKA did the experiments for Fig. 6A; and ECP did the experiments for Fig. 5. DJH helped XT and ECP on the experiments under anaerobic conditions. HL wrote the manuscript with input from all authors.
